# Emissions and Char Quality of Flame-Curtain "Kon Tiki" Kilns for Farmer-Scale Charcoal/Biochar Production

**DOI:** 10.1371/journal.pone.0154617

**Published:** 2016-05-18

**Authors:** Gerard Cornelissen, Naba Raj Pandit, Paul Taylor, Bishnu Hari Pandit, Magnus Sparrevik, Hans Peter Schmidt

**Affiliations:** 1 Norwegian Geotechnical Institute (NGI), Oslo, Norway; 2 Institute for Environmental Sciences (IMV), University of Life Sciences (NMBU), Ås, Norway; 3 Nepal Agroforestry Foundation (NAF), Kathmandu, Nepal; 4 Ithaka Institute for Carbon Strategies, Ancienne Eglise 9, Arbaz, Switzerland; 5 Department of Industrial Economics and Technology Management, Norwegian University of Technology, Trondheim, Norway; DOE Pacific Northwest National Laboratory, UNITED STATES

## Abstract

**Flame Curtain Biochar Kilns:**

Pyrolysis of organic waste or woody materials yields charcoal, a stable carbonaceous product that can be used for cooking or mixed into soil, in the latter case often termed "biochar". Traditional kiln technologies for charcoal production are slow and without treatment of the pyrolysis gases, resulting in emissions of gases (mainly methane and carbon monoxide) and aerosols that are both toxic and contribute to greenhouse gas emissions. In retort kilns pyrolysis gases are led back to a combustion chamber. This can reduce emissions substantially, but is costly and consumes a considerable amount of valuable ignition material such as wood during start-up. To overcome these problems, a novel type of technology, the Kon-Tiki flame curtain pyrolysis, is proposed. This technology combines the simplicity of the traditional kiln with the combustion of pyrolysis gases in the flame curtain (similar to retort kilns), also avoiding use of external fuel for start-up.

**Biochar Characteristics:**

A field study in Nepal using various feedstocks showed char yields of 22 ± 5% on a dry weight basis and 40 ± 11% on a C basis. Biochars with high C contents (76 ± 9%; n = 57), average surface areas (11 to 215 m^2^ g^-1^), low EPA16—PAHs (2.3 to 6.6 mg kg^-1^) and high CECs (43 to 217 cmol_c_/kg)(average for all feedstocks, mainly woody shrubs) were obtained, in compliance with the European Biochar Certificate (EBC).

**Gas Emission Factors:**

Mean emission factors for the flame curtain kilns were (g kg^-1^ biochar for all feedstocks); CO_2_ = 4300 ± 1700, CO = 54 ± 35, non-methane volatile organic compounds (NMVOC) = 6 ± 3, CH_4_ = 30 ± 60, aerosols (PM_10_) = 11 ± 15, total products of incomplete combustion (PIC) = 100 ± 83 and NO_x_ = 0.4 ± 0.3. The flame curtain kilns emitted statistically significantly (p<0.05) lower amounts of CO, PIC and NO_x_ than retort and traditional kilns, and higher amounts of CO_2_.

**Implications:**

With benefits such as high quality biochar, low emission, no need for start-up fuel, fast pyrolysis time and, importantly, easy and cheap construction and operation the flame curtain technology represent a promising possibility for sustainable rural biochar production.

## Introduction

Biochar, a heterogeneous substance rich in aromatic carbon and minerals, is produced by pyrolysis of sustainably obtained biomass under controlled conditions. Biochar has been suggested to be used as a soil amendment to improve crop productivity especially in weathered and eroded tropical soils [1–3]. While the production of biochar in modern industrial devices can be a highly controlled process with low noxious emissions and resulting in certifiable high quality biochar [4, 5], the technology has elevated costs of investment (> US$ 500,000) and maintenance leading to current market prices in the range of US$ 600–900 per ton of biochar [6–8]. In developing countries where most of the weathered tropical soils are found, biochar is not an option at these costs.

Many charcoal-containing Terra Preta soils in e.g. the Amazonas region, Germany, Australia, China and Scandinavia [9] prove, however, that ancient people must have known how to produce large quantities of biochar without the help of modern steel-based technology.

As charcoal was necessary to reach the temperature for iron ore melting, the history of civilization has been linked to charcoal production since the beginning of the Iron Age. For more than 3000 years most charcoal was and still is in many developing countries produced with inefficient and polluting methods since syngases with significant caloric value are released into the atmosphere. These include methane, carbon monoxide (CO) and aerosols (smoke; PM_2.5_ or PM_10_), nitrogen oxide and dioxide (NO and NO_2_, together NO_x_), as well as non-methane volatile organic matter (NMVOC), in addition to hydrogen [10]. Many of these gases are deleterious to human health, and/or they exacerbate anthropogenic radiative forcing. Cleaner but simple and accessible charcoal-making technologies are thus desirable.

Several traditional and low cost technologies to produce charcoal exist. They are either based on traditional methods practiced for centuries already or were adapted with more modern materials like mild steel to improve their efficiency. In most cases they are not used to produce biochar for agriculture but to produce charcoal for cooking or for export [11]. For tropical rural settings, the most important challenges have been to introduce a technology that is affordable and preferably free to farmers [12], as well as one that generates as low as possible gas and particle emissions.

The most important low-technology production methods for biochar include:

1. *Traditional earth mound or earth covered pit kilns* usually deliver good quality biochar though only high-value wood logs can be used as feedstock. The main environmental drawback is that toxic pyrolysis gazes are emitted unburned into the atmosphere generating significant gas emissions [10]. In addition yields are relatively low (10–20%) [10, 12] and the pyrolysis process is very slow, taking several days.
2. The development of the *Adam retort kiln and similar devices such as basic steel retort systems* introduced the partial afterburning of pyrolysis gazes. In these retort systems the feedstock wood can be mixed with dry biowaste materials like prunings, rice husks or maize cobs but a lot of valuable start-up wood is still needed [12, 13]. Such medium-scale improved retort technologies, where the pyrolytic gases are recirculated into the combustion chamber and combusted internally [14], produce around 75% lower deleterious gas emissions (mainly CO, CH_4_, aerosols) and higher conversion efficiencies of 30–45% than traditional systems. Energy contained in the recirculated carbon- and hydrogen rich syngases is thus used to sustain the pyrolysis process so that less heat from the endothermic pyrolysis reactions is needed to sustain the process [12, 13]. Moreover, the recirculation of pyrolytic gases leads to enhanced secondary char formation which also increases yield [15, 16].
3. Household-scale cooking stoves, so-called *TLUDs (Top-Lit Up-Draft stoves)* [17] can generate biochar while using the energy produced for cooking. Advantages include that they burn cleanly avoiding negative health effects due to indoor air emissions [18], can use various waste biomasses as feedstock and are fuel-efficient. Pyrolytic gases are mostly combusted in the flame front, reducing emissions of CO, CH_4_ and aerosols by around 75% [19, 20] compared to traditional cooking. Small-scale TLUDs may be applicable for horticulture or small kitchen gardens [21] but they generate too little biochar (0.5–1 kg per run for household devices and up to 10 kg for the bigger community stoves) to supply enough biochar for farming or selling as charcoal. In addition, the stove needs to be actively quenched after each cycle, which is impractical in daily use.

Thus the implementation of biochar into agricultural practice and the efficiency of the charcoal industry have been hindered by the absence of a low or zero-cost but clean charcoal-producing technology that would allow the on farm production of high-quality charcoal in sufficient amounts. A recent development has been the introduction of the *Kon-Tiki* flame curtain kiln, designed in 2014 in Switzerland and rapidly spreading since by open source technology transfer to farmers in more than 50 countries [22].

One run of a 2 m^3^ flame curtain kiln with an upper diameter of 2.4 m produces 500 kg of biochar (dry matter basis) and close to 2 MWh of heat from shrubs, husks, straw, prunings and other organic farm waste in about three hours needing one worker to maintain and control the process. In contrast to medium-sized retort kilns, no startup wood is needed for flame curtain kilns. The cost per kiln varies with design, construction material and country but is within a range of €30 (soil pit shield) to €5000. The cheapest way is a mere conically shaped soil pit which would essentially be for free.

In this paper, the gas and particle emissions of various flame curtain kiln designs were investigated, as well as the quality of the resulting biochars. To this end, 17 runs were performed with different feedstock mixtures in six different flame curtain kiln types, at Matathirta, a suburb of Kathmandu, Nepal. The basic feedstock was *Eupatorium adenophorum*, a very frequently occurring invasive forest shrub species that local people call “ban mara” (i.e. forest killer) [23] which is around 1–2 m high with stems up to 2 cm thick. Eupatorium was either pyrolyzed alone or blended with mixed firewood or rice husk. Gas and particle emissions (CO_2_, CH_4_, CO, NMVOC, aerosols/PM_10_, NO_x_) were determined, as well as biochar characteristics (elemental composition, specific surface area, polycyclic aromatic hydrocarbon (PAH) content, cation exchange capacity (CEC)). Thus, this paper provides important information on the performance and sustainability of a new, rapidly spreading biochar and charcoal making technology at an early stage.

## Materials and Methods

### Principle of the flame curtain kiln

The principle of the flame curtain pyrolysis consists of pyrolyzing biomass layer by layer in a conically formed metal kiln or soil pit (S1 Fig). A fire is started in the kiln, and the burning embers spread to form a first layer on the bottom of the kiln. A thin layer of biomass is then added on top of the embers, heats quickly and starts outgassing. The rising pyrolysis gas is caught in the flames and reacts with combustion air entering the kiln from the top. When ash appears on the outside of the carbonizing biomass, the next layer of biomass is homogenously spread on top. Convective and radiant energy from the flames above and from the hot pyrolyzing layers below heat the fresh biomass layer, which starts to pyrolyze [24].

The biochar below the upper pyrolysis layer is shielded from oxygen access by the fire curtain itself. The combustion zone thus forms a flame curtain that protects the underlying biochar from oxidizing and cleanly burns all pyrolysis smoke and gases as they pass through this hot fire front. It is important to spread each new biomass layer at the right time and rate determined by monitoring the flame, smoke and ash formation. Too much feedstock will smother the flame (producing smoke and gas emissions), and too little feedstock will not maintain a full curtain of flame to protect the biochar from oxidizing (forming ash) and to completely combust the pyrolysis gases (avoiding smoke). The manual layering of biomass is repeated until the metal kiln or soil pit is filled. The pyrolysis process is then actively ended by quenching with water or a nutrient solution (e.g., urine, dissolved fertilizer) or, where water is not easily available, by snuffing with a layer of soil (see S1 Fig for an illustration of the quenching and snuffing process).

The temperature in the main pyrolysis zone just below the flame curtain is 680°C to 750°C [22, 24] and cools down slowly below the main pyrolysis zone when new feedstock layers are added to 150–450°C depending on the duration of batch before final quenching. When snuffed with soil, biochar temperature may be maintained at above 400°C for more than 24h depending how tight the snuffing layer and kiln are.

### Kiln designs

Five different kiln designs (deep cone metal kiln, soil pit kiln, metal-shield soil pit kiln, all with a capacity of 60–130 kg feedstock per run, and small shallow octagonal kiln, shallow and deep pyramid kilns, all with a capacity of 15–25 kg feedstock per run) were tested with different feedstock and feedstock blending (wood, eupatorium shrubs, rice husks) producing between 120 to 800 l biochar per run. The essential difference between the kilns was the diameter, the outer angle and the material of the kiln (see Table 1 and S1 Fig).

**Table 1:** List over the experimental runs, feedstocks, masses, biochar yields (both as % of total mass and as % of C), biochar C, H, N contents, surface areas (SA), Cation Exchange Capacities (CEC) and PAH contents. CEC both for unwashed (including soluble ash, i.e., both exchangeable bases and soluble cations) and washed biochar (soluble ash removed, i.e., the "real" CEC), with the difference being the apparent CEC stemming from soluble cations in the ash ("CEC ash").

	Feedstock ratio			Quench	Biochar											
	Eupa-torium	Wood	Rice husk		C	H	N	Mass Yield	C yield	CEC Unwashed	CEC Washed	CEC Ash	SA	Total PAH	PAH excl. NAP^d^	BaP
	%	%	%		%	%	%	%	%	cmol_c_/kg	cmol_c_/kg	cmol_c_/kg	m^2^/g	mg/kg	mg/kg	mg/kg
All-steel deep octagonal																
	100	0	0	Water	77.0 ± 0.8	n.d.	n.d.	19	36		121		84.9		3.7	0.016
	80	20	0	Water	78.7	2.1	0.80	17	31		97					
BC_E-wood_^c^	50	50	0	Water	80.5	1.89	0.6	18	32		60		149^c^	2.3^c^		
Steel-shielded soil pit																
	100	0	0	Soil	71.2 ± 2.4	n.d.	n.d.	25	44	121	55	66	35.4		1.9	0.013
	80	20	0	Soil	88.8 ± 0.3	n.d.	n.d.	32	66	82	48	33				
	50	50	0	Soil	83.6	2.7	0.54	31	58	50	43	7				
Conical soil pit																
BC_E-soil_^c^	100	0	0	Soil	71.7	1.41	0.66	18	31	95	68	27	111^c^	6.6^c^		
	80	20	0	Soil	85.3 ± 2.1	n.d.	n.d.	27	54	63	55	8	74.6		2.0	0.037
	50	50	0	Soil	80.4	2.1	0.59	25	44	80	56	24				
All-steel shallow pyramidal and octagonal kilns																
Pyr 45° ^a^	100	0	0	Water	75.3 ± 2.3	1.3	1.04	21	39				215^c^	4.9^c^		
Pyr 45°	50	50	0	Water	74.1 ± 2.0	n.d.	n.d.	20	37		97					
Pyr 55°	100	0	0	Water	76.5 ± 0.2	2.0	0.72	17	32		101		72.9		4.2	0.020
Pyr 55°	100	0	0	Water	84.1	n.d.	n.d.	20	42		82					
Oct 55° ^b^	50	0	50	Water	54.7 ± 1.6	2.2	0.68	25	34				10.8		4.5	0.058
Pyr 45°	50	0	50	Water	55.0	n.d.	n.d.	25	34		45					
BC_E-met_ ^c^ Oct 55°	100	0	0	Water	72 ± 1.1	1.33	0.54	13	22		130					
Pyr 45° heat shield	100	0	0	Water	72.5 ± 1.8	n.d.	n.d.	27	49		217					

^a^ Pyramidal-shaped, angle 45 degrees.

^b^ octagonal-shaped, angle 60 degrees.

^c^ The biochars BC_E-wood_, BC_E-soil_ and BC_E-met_ were analyzed according to the EBC certificate;

^d^ PAH content excluding naphthalene.

### Moisture content

Prior to the startup of each run, the feedstock for pyrolysis was weighed. Moisture in the feedstock was measured with a *Voltcraft FM*-*300* Wood Humidity Meter at 1% accuracy. The Eupatorium contained 25% moisture, whereas the firewood and the rice husk contained 15% moisture. The mass and volume of the biochar were measured directly after water quenching or soil quenching. Dry mass of biochar was analyzed by drying at 110°C until mass equilibrium [12].

### Biochar characterization

Carbon content in feedstock (and char) was measured in triplicates on 100-mg samples that were combusted at 1030°C and analyzed in an element analyzer (Perkin-Elmer Optima 5300 DV Inductively Coupled Plasma Optical Emission Spectrometer (ICP-OES)). Wood feedstock was analyzed to contain 50.1% C, Eupatorium shrub 40.3% C, and in our parallel project in Tanzania rice husk was analyzed to contain 41.1% C, in accordance with literature values [25]. All biochars were characterized for cation exchange capacity by extraction with ammonium acetate at pH 7, both before and after washing with water for those samples where quenching was done with soil, and only after washing for the water-quenched samples [26]. Three biochars representing two different kiln types (soil pit kiln and metal cone kiln each 70°—1m50 diameter) and two feedstock (100% Eupatorium and 50:50 Eupatorium: hard wood) were analyzed by a EBC accredited laboratory following the EBC certification program and methods [4, 27]. Five example biochars were further analyzed for 15 individual PAHs by 36-h exhaustive toluene Soxhlet extraction according to published procedures [28, 29] and surface area by N_2_ adsorption at 77 K.

### Gas emission factors

The gases analyzed were CO_2_, CH_4_, non-methane volatile organic carbon (NMVOC), nitric oxides (NO_x_) and aerosols (total suspended particles, TSP, derived from PM_10_, for details see SI and [10]). Based on the measurements the value for total products of incomplete combustion (PIC) was given by summarizing the values for CO+NMVOC+CH_4_ and TSP (from PM_10_). A Microtector II 6460 was used to analyze carbon dioxide (CO_2_) and methane (CH_4_), both with a detection limit of 0.1% by infrared sensors and non-methane volatile organic components (NMVOC) with a detection limit of 0.1 ppm by photoionization detection (PID). The PID was calibrated using isobutene. Carbon monoxide (CO) and nitric oxide (NO) were analyzed with a Kigaz 300 flue gas analyzer by internal jacket type electrochemical sensors. Detection limits were 1 ppm for both sensors. For CO values above 8000 ppm the Kigaz instrument internally dilutes the gas stream to be able to measure concentrations up to 50 000 ppm. The instrument converts NO to generic nitric oxides (NO_x_) by applying a conversion factor of 1.03, thus assuming that 97% of NOx consists of NO. Particles in the form of PM_10_ were analyzed with a Thermo Scientific pdr-1500 instrument by use of photometric detection of particles (detection limit 0.1 μg/m^3^).

For conversion of concentration from mass units to molar ratios in the particle measurements, all particles were assumed to consist of elementary carbon. For subsequent conversion from TSP to total suspended particles (PM_10_) a conversion factor of 1.4 was used, thus assuming around 70% content of PM_10_ in the samples [12]. All sensors except the particle analyzer were protected by a 0.45μm particle filter that was changed regularly during measurements. Readings were taken as composite samples from the chimneys of the kilns during the pyrolysis process. Between three and ten readings were taken during the process depending on the duration of the charring. For further details and limits of detection, see S1 Description. In order to calculate the emission factors of the kilns the carbon balance method was utilized [10, 12, 30]. In this method, only the emission ratios between the gases are measured without the need to register the absolute mass of gases emitted. Instead, this mass is calculated by performing a carbon balance between the biomass entering the process and the biochar produced. From ten to twenty single-point ratios, time-weighted average values were calculated. Net molar component-to-CO_2_ emission ratios for the measured gases and TSP (from PM_10_) for the flame curtain runs were 0.02 for CO, 0.02 for CH_4_, 0.001 for NMVOC, 0.01 for TSP and 0.0001 for NO_x_. These ratios were used to calculate the emission factors in g per kg biochar produced. Details of the calculation method can be found in ref. [12] and are presented again in S2 Description.

### Statistics

A two sample t-test with nonsimilar variance using R was used to test for effects of kiln type on gas emission factors (CO_2_, CO, VOC, CH_4_, TSP, PIC and NO). The emission factors for the flame curtain kilns were compared to those of traditional kilns and retort kilns measured in different countries and for different feedstocks but with exactly the same instruments [12]. Differences with p-values < 0.05 were considered significant.

## Results and Discussion

### Biochar yields

Biochar yields were 22 ± 5% on a dry weight basis and 40 ± 11% on a C basis (Table 1). This is in the same order of magnitude as other high temperature (700°C) pyrolysis systems [31–34]. It is also in the same order of magnitude of traditional low-temperature kilns but lower than low temperature retort kilns (typically around 30–40% on a dry weight basis [12, 13]).

Yields were significantly higher for the soil-quenched kilns (26 ± 5%) than for the water-quenched kilns (20 ± 4% including the rice husk/eupatorium runs, 19 ± 5% excluding these) (Table 1), mainly because of the dissolution and wash off some of the ashes in the water-quenched kilns and probably also because of the inevitable mixing of the biochar with soil minerals from the kiln and snuffing layer. Biochar yields were rather variable (13 to 32%), probably due to variation in operation conditions (frequency of biomass addition) and meteorological conditions (wind, air moisture, temperature) but also reflect "real-world" conditions where biochar yields with this method can be expected to be equally variable. Further factors influencing the biochar yield in flame curtain kilns are water content, particle size and bulk density of the feedstock. The higher the water content of the feedstock, the more combustion energy is needed to evaporate the water and to heat the feedstock to pyrolysis temperatures above 300–400°C. This leads to longer exposure times of feedstock material to the reduced combustion air at the kiln surface, which causes more surface carbon to oxidize and results in higher ash content and lower biochar carbon yield. Equally, the duration of complete pyrolysis of the core of larger diameter wood pieces is much longer than for higher surface low diameter feedstocks like grain husks (rice husks) or shrub twigs (eupatorium). Such differences in pyrolysis duration explain higher carbon losses and thus lower yields of wood logs compared to twigs, straw or husks.

### Char characteristics

C contents of the chars were 75.5 ± 9% (n = 37; Table 1), the lowest value being for the rice husk / Eupatorium 50/50 mixed feedstock runs (54–55%), due to the high inorganic (silica) content of the rice husk [35]. H contents of nine example biochars were 1.85 ± 0.5%, N contents were 0.69 ± 0.16% and C/N ratios were 118 ± 28. The three EBC tested biochars have molar H:C_org_ ratios of 0.22 to 0.28 and molar O:C_org_ ratios of 0.04 to 0.07 confirming the high aromaticity expected for biochars made at temperatures around 700°C [36]. Surface areas of most biochars were in the range of 100–200 m^2^/g (Table 1) which is in agreement with other biochars produced with industrial technology at temperatures of 600° to 750°C [37].

Cation Exchange Capacities (CECs) of 15 biochars were 40–130 cmolc/kg, with one char even showing CEC above 200 cmolc/kg, which is on the high end of literature values for field-made biochars [26, 38, 39], indicating that the biochars probably have good nutrient-holding characteristics [26, 40]. For the soil-quenched chars, up to half of the "apparent" CEC for unwashed chars actually stemmed from soluble base cations in the ashes (Table 1).

Looking more closely at the three more completely characterized biochars (Table 2), the most apparent difference is the ash content being higher in both eupatorium biochars (BC_E-met_: 21.9% and BC_E-soil_: 19.9%) compared to the eupatorium-wood biochar (BC_E-wood_: 10.2%). This can be explained by the higher mineral content of eupatorium shrubs compared to hard wood and is confirmed by the much higher silica (34,000/34,000 vs 5400 g kg^-1^), iron (6,000/3,700 vs 950 g kg^-1^) and potassium (28,000/36,000 vs 19,000) content of the pure eupatorium chars. The nutrient contents further differed slightly between the two eupatorium chars which can be explained by the fact that the metal cone biochar was water quenched and lost a higher portion of soluble minerals while the concentration of some less soluble minerals increased compared to the soil snuffed biochar. This is illustrated most clearly by the highly soluble Na which was 5.5 times lower in the water quenched BC_E-met_ (520 mg/kg) than in the soil snuffed BC_E-soil_ (2900 mg/kg). The higher mineral content of both pure eupatorium chars is probably also the reason for the higher pH (9.8 / 9.6) compared to the eupatorium-wood char (8.7) which had also been water quenched.

**Table 2:** Analyses of three biochars made in three different kilns and with two different feedstocks. Analyzed by an EBC accredited laboratory following the EBC biochar analytical methods [4, 27] and compared to the EBC thresholds for premium and basic biochar quality.

Biochar name		BC_E-met_	BC_E-soil_	BC_E-wood_		
Kiln		60°—1.1 m steel	70°—1.5m soil pit	70° 1.5 m steel	EBC—threshold	
Biomass		Eupatorium	Eupatorium	Eupatorim—Wood (50:50)	premium	basic
Density	kg m^-3^	120	n.d.	n.d.		
Specific surface (BET)	m^-2^ g	215	149	111		
Ash 550°C	mass-%	21.9	19.9	10.2		
Hydrogen	mass-%	1.33	1.41	1.89		
Carbon	mass-%	72	71.7	80.5		
Nitrogen	mass-%	0.54	0.66	0.6		
Oxygen	mass-%	4.0	6.2	6.7		
Carbonate CO2	mass-%	2.24	1.3	1.81		
Organic carbon	mass-%	71.4	71.3	80.0	> 50	> 50
H/C org. (molar)		0.22	0.24	0.28	< 0.7	< 0.7
O/C (molar)		0.042	0.07	0.06	< 0.4	< 0.4
pH		9.8	9.6	8.7		
Electric conductivity	μS cm^-1^	9090	n.d.	n.d.		
Salt content	g kg^-1^	53.7	n.d.	n.d.		
Phosphorous	mg kg^-1^	3700	4600	3800		
Magnesium	mg kg^-1^	12000	4100	3800		
Calcium	mg kg^-1^	17000	15000	26000		
Potassium	mg kg^-1^	28000	36000	19000		
Sodium	mg kg^-1^	520	2900	860		
Iron	mg kg^-1^	6000	3700	950		
Silica	mg kg^-1^	34000	34000	5400		
Sulfur	mg kg^-1^	860	1800	1000		
Lead	mg kg^-1^	< 2	4	< 2	< 120	< 150
Cadmium	mg kg^-1^	< 0.2	< 0.2	< 0.2	< 1.5	< 1.5
Copper	mg kg^-1^	30	19	16	< 100	< 100
Nickel	mg kg^-1^	5	14	12	< 30	< 50
Mercury	mg kg^-1^	< 0.07	< 0.07	< 0.07	< 1	< 1
Zinc	mg kg^-1^	120	61	39	< 400	< 400
Chromium	mg kg^-1^	7	15	14	< 80	< 90
Boron	mg kg^-1^	74	10	< 1		
Manganese	mg kg^-1^	210	300	200		

The heavy metal contents were all low compared to the EBC thresholds indicating clean biomass feedstock. Interestingly, the zinc content of the pure eupatorium chars was comparably high, which could indicate zinc accumulation by the Eupatorium plants, as other sources of contamination can probably be excluded.

The most toxic compound among the PAH-16 used as benchmarks by the environmental authorities in many countries is benzo(a)pyrene. Concentrations of benzo(a)pyrene were 0.01–0.06 mg/kg (Table 1), well below the Norwegian maximum tolerable risk (MTR) level for soils where 95% of art diversity is protected (0.5 mg/kg)[41]. In addition, PAHs in biochar are only very sparingly bioavailable, often less than 1% [28]. Due most probably to the optimized out-gassing under the fire front the PAH EPA16 contents were low (2.3 to 6.6 mg kg^-1^). However, while both water quenched metal cone biochars would qualify for EBC premium quality (< 4 ±2 mg kg^-1^), the soil snuffed biochar would only entitle for basic quality (< 12±4 mg kg^-1^). It can be assumed that the hot water vapor that penetrates from bottom to top through the biochar layers during the water quenching process has an activating effect and may expulse PAH containing gases out of the biochar pores [42]. This activating and tar reducing effect can also be seen in the nearly 50% higher specific surface area of the water quenched eupatorium char (215 m^2^ g^-1^) compared to the soil snuffed char (149 m^2^ g^-1^).

The thermogravimetric analysis (TGA) (S2 Fig) of BC_E-soil_ and BC_E-wood_ showed that for both biochars the highest treatment temperature (HTT) was in between 680° and 750°C. The curb between 150° and 550°C showed a rather regular continuum of volatile organic carbon (VOC) release indicating a rather complete pyrolysis (no uncharred particles) and homogenous cooling at the end of the pyrolysis process. Interestingly, the pure eupatorium biochar has slightly lower VOC content (64% vs 70% at HTT) probably due to the smaller particle size of the eupatorium feedstock and thus faster heat conduction, faster pyrolysis and better vapor penetration during water quenching.

Overall, the three representative biochars produced in flame curtain kilns were of high quality comparable with high-tech produced higher temperature biochars [34, 43] and all qualifying for the EBC certificate which is the baseline for authorization to use biochar as soil amendment in e.g. Switzerland and Austria. Moreover, BC_E-met_ was already tested in an agronomic field trial in Nepal and proved its plant growth enhancing potential by increasing the pumpkin yield fourfold when blended with cow urine and compost and more than doubled when blended only with compost both compared to the control which was amended only with compost and cow urine [24].

### Gas emission factors

Emission factors were, in g per kg biochar produced: CO 54 ± 35, CH_4_ 30 ± 59, TSP 11 ± 15, NMVOC 6 ± 3, NO_x_ 0.4 ± 0.3, and total products of incomplete combustion, PIC, 100 ± 83. These data are based on 17 runs of 10 to 15 data points each, totaling around 250 individual measurements per gas/aerosol. The high standard deviations thus do not reflect a lack of data but rather a high variability of gas emissions during individual kiln runs. This variability is caused by variations in burning conditions during the individual runs: e.g. if the flame curtain is interrupted by putting on too much feedstock, pyrolysis gases are not completely combusted and spikes in gas emissions are observed. In addition, the above-mentioned variations in biochar yield influence the emission factors in g per kg biochar. Especially the methane emission data (Table 3) had large standard deviations: methane concentrations were mostly below the limit of detection of 0.1% (around 10 g/kg biochar), whereas they occasionally leaped up to 1–3% (100–300 g/kg char). Such spikes coincided with events where much of the flame curtain was absent due to feeding with too much feedstock, underscoring that the flame curtain is pivotal to sustain low emissions.

**Table 3:** Emission factors (g/kg charcoal) of CO_2_, CO, CH_4_, TSP [aerosols, from particulate matter < 10 μm (PM_10_)], non-methane volatile organic carbon (NMVOC), and the sum of nitrogen oxide and nitrogen dioxide (NO_x_), as well as the sum of all products of incomplete combustion, PIC (all gases except CO_2_). Average values per flame curtain kiln type and per feedstock, and kiln literature values (traditional non-improved kilns, retort kilns with syngas circulation and combustion, TLUDs).

		n^a^	CO_2_	CO	NMVOC	CH_4_	TSP	PIC	NO
Per flame curtain kiln type									
All-Steel deep octagonal	this study	n = 3	5600 ± 700	38 ± 20	6 ± 2	57 ± 52	22 ± 28	123 ± 82	0.3 ± 0.1
Steel-shield Soil pit	this study	n = 3	2300 ± 800	23 ± 28	5 ± 5	14 ± 20	9 ± 7	51 ± 31	0.3 ± 0.2
Soil pit	this study	n = 3	3800 ± 1300	36 ± 40	8 ± 1	32 ± 44	20 ± 24	97 ± 108	0.8 ± 0.7
shallow steel pyramidal and octagonal	this study	n = 10	4700 ± 800	73 ± 31	5 ± 3	26 ± 75^b^	5 ± 4	108 ± 93	0.32 ± 0.12
Per feedstock type									
100% Eupatorium	this study	n = 9	4600 ± 2100	74 ± 34	6 ± 3	60 ± 90^b^	11 ± 16	151 ± 109	0.4 ± 0.2
80% Eup, 20% wood	this study	n = 3	3400 ± 2300	23 ± 26	5 ± 3	28 ± 34	23 ± 27	79 ± 89	0.1 ± 0.2
50% Eup, 50% wood	this study	n = 3	3900 ± 2000	13 ± 4	9 ± 1	13 ± 21^c^	9 ± 7	43 ± 25	0.7 ± 0.6
50% Eup, 50% Rice husk	this study	n = 2	3810 ± 50	47 ± 16	3.0 ± 0.2	0	3 ± 2	52 ± 19	0.260 ± 0.002
Kiln literature									
Traditional kiln	Ref. [10, 12]^d^	n = 8^e^	2375	351	53	49	19	472	2.2
Retort kiln	Ref. [10, 12]^d^	n = 5^e^	2602	148	7	35	11	202	1.7
TLUD	Ref. [20]	n = 5^e^	n.r.	94	274	40	7	415	0.0
High-tech large-scale reactor	Ref. [44]		3010	3·10^−7^	0	0	0.05	0.05	0.7

^a^ n is number of datasets (time series during one kiln run). Each dataset consists of 10–15 measurements. Thus, the total number of measurements is 20 to 150.

^b^ large std since value is dominated by one large value of 238 g/kg char.

^c^ large std since value is dominated by one large value of 37 g/kg char.

^d^ average of two literature datasets where each data set was given equal weight.

^e^ one dataset per kiln type.

**Fig 1:**
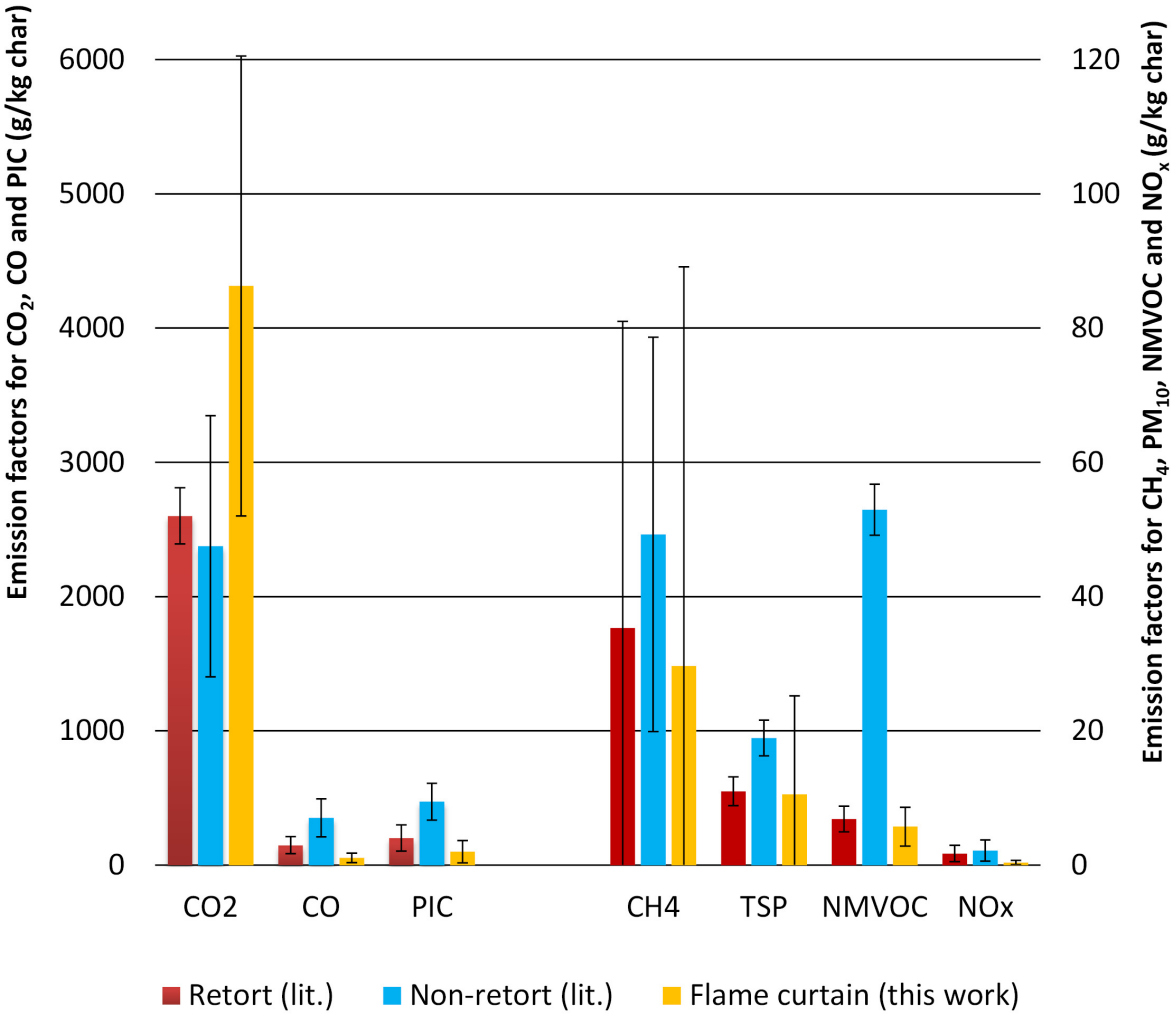
Emission factors for CO_2_, CO, CH_4_, TSP (aerosols, derived from PM_10_ as described in the methods, non-methane volatile organic carbon (NMVOC), and the sum of nitrogen oxide and nitrogen dioxide (NO_x_), as well as the sum of all products of incomplete combustion, PIC (all C-containing gases except CO_2_). Flame curtain: based on 17 runs of 10 to 15 measurements each done within the present study. Retort and non-retort kilns: average values from refs. [10, 12]. Error bars represent standard deviations in 50 to 250 individual measurements.

Fig 1 compares the average emission factors for the flame curtain kilns (n = 17) with values that were previously measured for traditional and retort kilns. For the comparison to retort kilns only values from Sparrevik et al. [12] were used because these measurements were carried out with exactly the same equipment and measuring methodology and because we dispose of the complete series of data for these measurements. For the comparison to traditional kilns, data from [12] and [10] were used. Overall, the data were based on eight runs for traditional kilns, and five runs for retort kilns.

The flame curtain kilns had significantly lower emissions of CO and NO_x_ (54 ± 35 and 0.4 ± 0.3 g/kg biochar, respectively) than traditional or retort kilns (CO: 351 ± 141 and 148 ± 64 g/kg biochar, respectively; NO_x_: 2.0 ± 1.6 and 1.7 ± 1.0 g/kg biochar, respectively). The total products of incomplete combustion (PIC) emissions of the flame curtain kilns were significantly lower than those of non-retort and retort kilns. Non-methane volatile organic carbon (NMVOC) emissions were significantly lower for flame curtain kilns (6 ± 3 g/kg biochar) than for traditional kilns (53 ± 4 g/kg biochar). Methane and TSP emissions were not significantly different between the flame curtain, traditional and retort kilns. CO_2_ emissions were significantly higher for the flame curtain kilns than for retort or traditional kilns, which is a direct consequence of the slightly lower yields and lower non CO_2_-emissions obtained in flame curtain kilns. CO_2_ is the lowest caloric and least climate hazardous emission product of biomass combustion and a measure of the completeness of the combustion of pyrolytic gases. PIC, the sum of all C-containing products of incomplete combustion, is dominated by CO (around 30 to 70%), and thus PIC could be lower for flame curtain kilns than for retort and traditional ones, even though TSP (< 20% of the total PIC) was not.

In flame curtain pyrolysis the combustion of the main pyrolysis gases appears to be fairly complete due to efficient and turbulent mixing of these gases with combustion air above the pyrolysis zone. However, the heat and combustion dynamic is apparently not sufficient to completely combust less inflammable aerosols (TSP). For that reason TSP rates were comparable to retort kilns while the emission of the more ignitable pyrolysis gases like CO, and NMVOC was significantly lower.

The currently measured emission factors were comparable to literature values for TLUD stoves (Table 3), with the exception of NMVOC, where literature values are approximately one order of magnitude higher than the values for flame curtain pyrolysis. The similarity in gas and TSP emission factors between flame curtain kilns and TLUD stoves was expected because of the similar principle of pyrolysis gas combustion, where pyrolytic gases are formed below a flame, carried upwards by the up-draft and subsequently combusted in the flame, suppressing the emission of combustible gases and particles such as CO, methane, NMVOC and aerosols.

The lower yields, higher CO_2_ emissions and lower CO emissions for flame curtain kilns compared to traditional or retort kilns are explained by the principle of the open flame curtain: close to the high temperature of the open flames more feedstock gasifies and these pyrolytic gases combust more completely which results in lower yields, higher CO_2_ emissions and lower combustible emissions like CO, CH_4_ and others.

### Various flame curtain kiln subtypes

Differences between the various subtypes of flame curtain kilns or various feedstocks were non-significant in all cases except CO_2_ emissions from steel-shielded soil pit kilns (2300 ± 800 g/kg biochar) being lower than those of all-steel deep cone kilns (5600 ± 700 g/kg biochar)(Table 3). This result is encouraging in the sense that simple conically shaped soil pit flame curtain kilns, if they are operated properly, result in biochar yields, C contents and gas / aerosol emissions that are similar to those of the all-steel deep conical flame curtain kilns. This implies that high-quality biochar can be made in a sustainable manner without investing more than for the labor involved in digging out the soil pit, drying the feedstock and carrying out the pyrolysis.

For the two runs done with 50% rice husk and 50% Eupatorium, emission factors did not significantly differ from those for eupatorium or eupatorium/wood mixtures (Table 3). It should be noted though that the timing of the layer placement during pyrolysis is more crucial for the rice husk than for the other feedstocks because if too much of the low-densityrice husks are added too quick and/or at once, the flames are snuffed which leads to higher emissions especially of methane and aerosols. Since the performed rice husk runs were executed by a skilled operator, such emissions were not observed here.

### Implications

In Table 4, the advantages and disadvantages of various medium-size kiln types are compared. The biochar yield of 22 ± 5%, which is somewhat lower than that of retort kilns [12, 13], is a disadvantage of flame curtain kilns. This is not a significant hindrance in the case of biochar for soil amendment made from low-value organic residues like shrubs, straw and husks which are materials that cannot be pyrolysed in such retort system without a large portion of valuable fire wood. However, it is an important factor to consider in the case of charcoal making from high-value wood for cooking purposes, where yields need to be high in order to reduce deforestation and increase the economic value of the charcoal making activity.

**Table 4:** Advantages and disadvantages of various medium-size kiln types.

	Application	Main advantages	Main disadvantages
Biochar-generating TLUD cookstove	Kitchen gardens, cooking purposes	Energy for cooking, Saving firewood, Low gas emission factors	Too small to generate larger amounts of biochar
Traditional kilns	Agriculture, charcoal making	Familiarity, Low investment cost, Complete pyrolysis of thicker logs	High gas emission factors, Slow (4 days)
Retort kilns	Agriculture (possibly + energy), charcoal/briquette making	Lower emissions than traditional kilns, High biochar yield, Energy generation possible with pyrolysis heat, Complete pyrolysis of thicker logs	High investment cost, Startup wood required, Complicated construction and operation, Slow (2 days)
Flame Curtain Kilns	Agriculture + heat, charcoal making (small logs)	Relatively low emissions esp. of CO, No startup wood required, Easy to construct and operate, Fast (3 hours for 1 m^3^ biochar), Low to zero investment cost, Heat recovery	Relatively low biochar yield (charcoal making), Incomplete pyrolysis of thick logs
Power-generating systems	Energy + agriculture, briquette making	Power generation, Negligible emissions	Relatively high investment cost, Low caloric content of briquettes

The flame curtain kiln offers multiple advantages:

1. gas and aerosol emissions are relatively low (for CO even lower than those of retort kilns) compared to other small scale biochar and charcoal production technologies but not to large-scale processes (Table 3);
2. no wood is required for startup;
3. construction and operation is much easier and more economic compared to retort kilns;
4. pyrolysis is much faster (hours) than in most traditional and retort kilns (days). The process might actually be too fast for the complete pyrolysis of thick wood logs in shallow kilns when thinner materials are mixed in; in case of charcoal making from wood logs, it is advised to use well-insulated deep cone kilns, to use only wood as feedstock, to finalize with thinner branches at the top and snuff with soil or rather iron lid instead of quenching with water;
5. heat from pyrolysis gas combustion can easily be recovered for drying, distillation, hot water production or cooking;
6. investment costs are low (for the steel deep cone kilns) to negligible (for the conically shaped soil pit kilns). The last argument might be decisive for tropical farmers on the poorest soils where biochar possibly has the strongest positive agricultural effects: as these farmers need to sustain on meager yields grown on these difficult soils, they often do not have the resources to invest in novel technologies. In the case of charcoal making for cooking purposes, the flame curtain kilns are certainly more sustainable than the also free earth-mound kilns, because of the advantages mentioned above especially the lower gas/aerosol emissions.

The quality of the flame curtain kiln biochars was good with regard to all relevant parameter for EBC and IBI certification and showed further high CEC and SSA values (Tables 1 and 2). Pyrolysis temperatures of the flame curtain kilns (700°C) are higher than those of traditional or retort technologies (400–500°C)[10, 12], and this results in a more porous and more condensed biochar [45]. Higher porosity certainly implies stronger contaminant immobilization [46] and probably also higher nutrient retention [47]. More condensed higher-temperature biochars exhibit higher H/C_org_ ratios which have been related to relatively strong N_2_O emissions reductions upon their amendment to soil in a recent meta-analysis [48]. Finally, in another meta-analysis higher-temperature chars have tentatively been associated with negative priming, i.e., increases in soil organic matter upon the amendment of biochar to soil [49]. Overall, in many cases the high-temperature flame curtain chars can be expected to be of higher quality than lower-temperature ones made by traditional technologies, depending on the purpose the respective biochar or charcoal is intended for.

## Conclusion

The Kon-Tiki flame curtain pyrolysis is a new type of low cost biochar and charcoal production technology with pyrolysis gas combustion. It can easily be built and used by farmers both in the developed and developing world. It was shown that the quality of biochar produced from various feedstocks complies with international quality standards like IBI and EBC. Gas and aerosol emissions were very low compared to all other low cost and traditional charcoal and biochar production devices.

## Supporting Information

S1 Description Gas Analyses. Experimental details of gas emission analyses.
(DOCX)

S2 Description Carbon balance and emission factors. Carbon balance and emission factors: accurate description of the calculation of carbon balance and gas emission factors.
(DOCX)

S1 Fig Kiln types. Overview of kiln types tested in this paper.
(DOCX)

S2 Fig TGA analyses. TGA analyses of two representative biochars (BC_E-soil_ and BC_E-wood_). Temperature was ramped from 25 to 950°C in 2 hours. "Gewichtsverlust" is loss of weight (both rate and overall loss), "Zeit" is time.
(DOCX)
