# Supplementary material for: Emissions and Char Quality of Flame-Curtain "Kon Tiki" Kilns for Farmer-Scale Charcoal/Biochar Production
Source: PLoS One. 2016 May 18;11(5):e0154617. doi: 10.1371/journal.pone.0154617 (PMC4871524; doi:10.1371/journal.pone.0154617)
Supplement: S1 Description — Experimental details of gas emission analyses. (DOCX) [file pone.0154617.s001.docx]

**S1 Description. Gas Analyses**. Experimental details of gas emission analyses

A Microtector II 6460 was used to analyze carbon dioxide (CO_2_) and methane (CH_4_), both with a detection limit of 0.1% by infrared sensors and non-methane volatile organic components (NMVOC) with a detection limit of 0.1 ppm by photoionization detection (PID). The PID was calibrated using isobutene. Carbon monoxide (CO) and nitric oxide (NO) were analyzed with a Kigaz 300 flue gas analyzer by internal jacket type electrochemical sensors. Detection limits was 1 ppm for both sensors. For CO values above 8000 ppm the Kigaz instrument internally dilutes the gas stream to be able to measure concentrations up to 50 000 ppm. The instrument converts NO to generic nitric oxides (NO_x_) by applying a conversion factor of 1.03, thus assuming that 97% of NOx consists of NO. Particles in the form of PM_10_ were analyzed with a Thermo Scientific pdr-1500 instrument by use of photometric detection of particles (detection limit 0.1 μg/m^3^). In fact the particles below 2.5 µm are the most carcinogenic to humans (Smith and Mehta, 2003). PM_10_ and PM_2.5_ have found to be well-correlated in some studies (Wang et al., 2006) but less so in others (Castillejos, 2000). In our case, we selected PM_10_ over PM_2.5_ since we then could avoid cyclones and pre-filtration, which may introduce unnecessary measure errors when working with direct measurements of exhaust gases.

For conversion of concentration from mass units to molar ratios in the particle measurements, all particles were assumed to consist of elementary carbon. For subsequent conversion from TSP to total suspended particles (PM_10_) a conversion factor of 1.4 was used, thus assuming around 70% content of PM_10_ in the samples (Schikowski et al., 2005). Based on the measurements the value for products of incomplete combustion (PIC) were given by summarizing the values for CO+NMVOC+CH_4_ and TSP. All sensors except the particle analyzer were protected by a 0.45μm particle filter that was changed regularly during measurements. Polytetrafluoroethylene (PTFE) tubing was used for sampling. Readings were taken as composite samples from the chimneys of the kilns during the pyrolysis process. Between three and ten readings were taken during the process depending on the duration of the charring. The samplings always included start-up and operational mode (retort mode when relevant). The cooling processes were not sampled since hardly any gases are emitted from the material during this period and thus their inclusion would result in skewed data. For all gases and readings, a molar ratio between the component and CO_2_ was calculated. From these single-point ratios, a time-weighted average (TWA) was calculated between each subsequent measurement point to be representative for this specific period of the process. The different portions were then integrated over the whole process period and a grand mean value representative for the whole carbonization process was calculated. Since the process is proceeding in different stages (including switching from non-retort to retort mode in the retort kilns) TWA is better representative for the process than the use of geometric mean values.
