# Supplementary material for: Emissions and Char Quality of Flame-Curtain "Kon Tiki" Kilns for Farmer-Scale Charcoal/Biochar Production
Source: PLoS One. 2016 May 18;11(5):e0154617. doi: 10.1371/journal.pone.0154617 (PMC4871524; doi:10.1371/journal.pone.0154617)
Supplement: S2 Description — Carbon balance and emission factors: accurate description of the calculation of carbon balance and gas emission factors. (DOCX) [file pone.0154617.s002.docx]

**S2 Description. Carbon balance and emission factors**. Carbon balance and emission factors: accurate description of the calculation of carbon balance and gas emission factors.

In order to calculate the emission factors of the kilns the widely used carbon balance method was utilized (Bailis et al., 2003; Pennise et al., 2001; Zhang et al., 2000). In this method, only the emission ratios between the gases are measured without the need to register the absolute mass of gases emitted. Instead, this mass is calculated by performing a carbon balance between the biomass entering the process and the biochar produced. Thus, the difference in carbon was assumed equal to the mass of carbon in the emitted gases. The molar ratios were then used to calculate the distribution of carbonaceous gases in the emitted smoke. For open systems like the present ones, the carbon balance method is preferable over absolute measurements of gas composition because controlling all gases escaping from the process is challenging.

Adapted from Zhang et al (Zhang et al., 2000), the mass balance of carbon in feedstock combustion process can be described as follows;

$C_{feedstock}-C_{char}=C_{{CO}_{2}}+C_{CO}+C_{{CH}_{4}}+C_{NMVOC}+C_{TSP}$ (1)

where C_feedstock_ is the carbon content in the biomass feedstock and C_char_ is the carbon content in the processed biochar material on the left side of equation (1). On the right side is the sum of all combustion gases containing carbon.

Rearranging eq. (1) yields;

$\frac{C_{feedstock}-C_{Char}}{C_{{CO}_{2}}}=1+\frac{C_{CO}}{C_{{CO}_{2}}}+\frac{C_{{CH}_{4}}}{C_{{CO}_{2}}}+\frac{C_{NMVOC}}{C_{{CO}_{2}}}+\frac{C_{TSP}}{C_{{CO}_{2}}}$ (2)

K can be defined as the sum of all emission ratios of the components to CO_2_

$K=\frac{C_{CO}}{C_{{CO}_{2}}}+\frac{C_{{CH}_{4}}}{C_{{CO}_{2}}}+\frac{C_{NMVOC}}{C_{{CO}_{2}}}+\frac{C_{TSP}}{C_{{CO}_{2}}}$, (3)

Subsequently 1+K can be defined to represent all products of pyrolysis including CO2.

The emission factor of CO_2_ on a carbon basis is defined as mass of emissions pr. mass of char produced (m_char_):

$E_{{m,CO}_{2}-C}=\frac{C_{{CO}_{2}}}{m_{char}}=\frac{C_{feedstock}-C_{char}}{\left( 1+K \right)m_{char}}$ (4)

By solving equation (4), the mass of CO_2_-C can be calculated and converted to CO_2_ by using the C/CO_2_ molar ratio. The other gases of interest can be found by their respective molar ratios to CO_2_-C.

After the calculation of the mass of all gaseous components, the emission ratio can be related either to the amount of char or to the amount of C produced. In the present study, we did not achieve significant amounts of visibly identifiable brads or ash (measured in a previous study with the same biochars showing around 10% ash (Hale et al., 2013)). Bio oil was attempted to be collected on surfaces as lids and drums after pyrolysis but the amounts were insignificant (<1% of the biochar mass produced). The emissions factors were therefore solely based on the weighing of all the produced material, which we defined as biochar.

The collection of gas emission data under rural conditions and at different field sites in various countries is a time-consuming and difficult process (Pennise et al., 2001). Even though multiple measurements of gases during the individual runs substantiated a correct representation of the emissions in that specific run, uncertainties may still be present since; i) kilns are different in construction, ii) a wide array of feedstocks can be used, and iii) the kilns were operated by local people using their operational practices. We addressed this by pooling the data together into two kiln types (retort and non-retort) and applying statistical analysis to conclude on the validity of the results especially sensitivity to use of different feedstocks. A two sample t-test using the statistical package SPSS statistics version 21 was used to test for effects of kiln type (retort and non-retort) on; i) biochar yield, ii) molar ratios and finally on iii) emission factors (CO_2_, CO, VOC, CH_4_, TSP, PIC and NO).
