# Supplementary material for: Emissions and Char Quality of Flame-Curtain "Kon Tiki" Kilns for Farmer-Scale Charcoal/Biochar Production
Source: PLoS One. 2016 May 18;11(5):e0154617. doi: 10.1371/journal.pone.0154617 (PMC4871524; doi:10.1371/journal.pone.0154617)
Supplement: S1 Fig — Overview of kiln types tested in this paper. (DOCX) [file pone.0154617.s003.docx]

**S1 Fig. Kiln types.** Overview of kiln types tested in this paper.

| 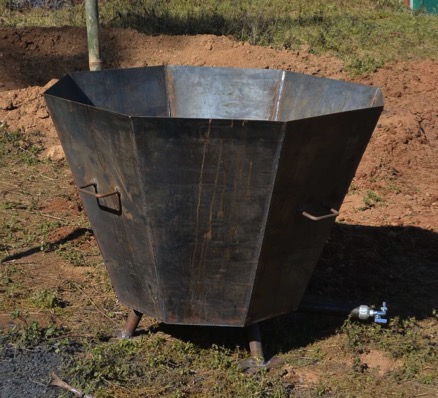 | All-steel deep octagonal kiln  Octagonal deep cone kiln without rim shield.  Upper long diagonal: 1500 mm  Lower long diagonal: 820 mm  Depth: 980 mm  Outer angle: 70° |
| --- | --- |
| 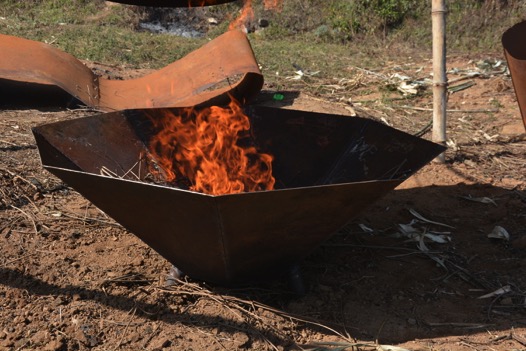 | All-steel shallow octagonal kiln Oct 55°  Octagonal shallow cone kiln without rim shield.  Upper long diagonal: 1131 mm  Lower long diagonal: 381 mm  Depth: 400 mm  Outer angle: 55° |
| 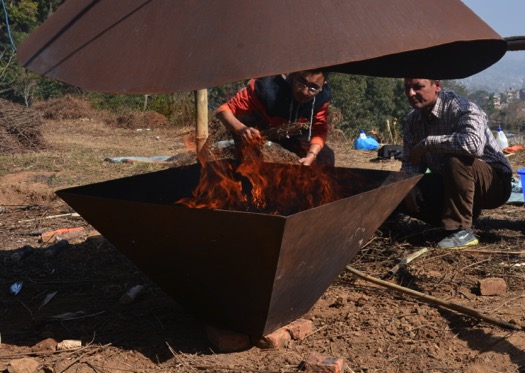 | All-steel pyramid kiln Pyr 55°  Upper long diagonal: 1100 mm  Lower long diagonal: 382 mm  Depth: 400 mm  Outer angle: 55° |
| 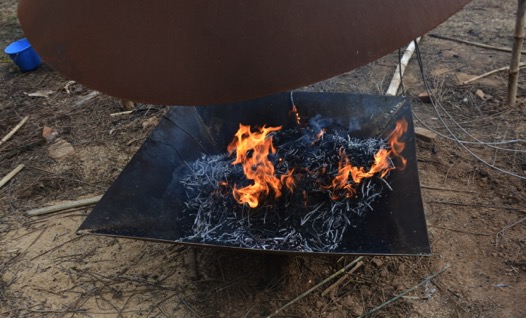 | All-steel shallow pyramid kiln Pyr 45°  Upper long diagonal: 1740 mm  Lower long diagonal: 382 mm  Depth: 480 mm  Outer angle: 45° |
| 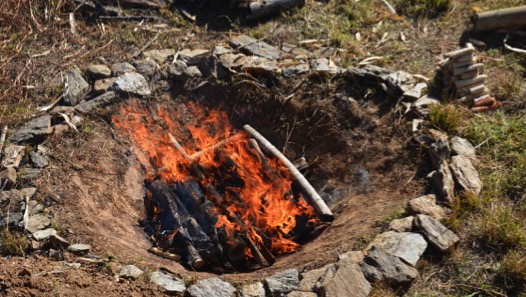 | Conical soil pit  Upper diameter: 1500 mm  Lower diameter: 800 mm  Depth: 900 mm  Outer angle: 60-70° |
| 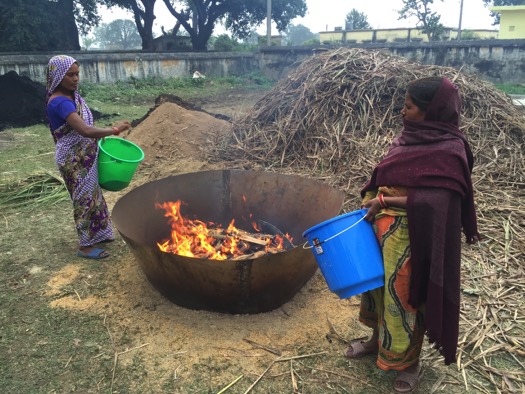 | Steel-shielded soil pit  Upper diameter: 1500 mm  Lower diameter: 800 mm  Depth: 900 mm  Outer angle: 70° |
| 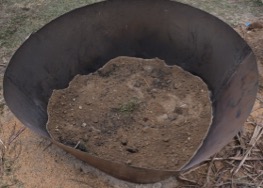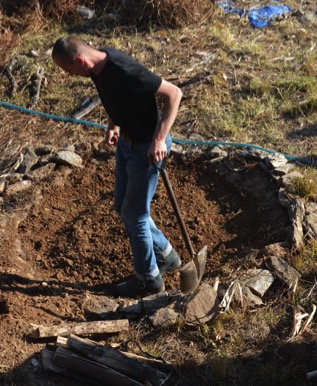 | Snuffing of soil pit kiln with soil, pressed with feet and shovel to make it air-tight. |
| 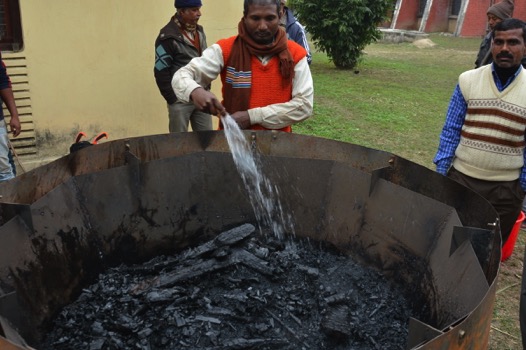 | Quenching with water or nutrient liquids    If water pressure is sufficient, the water is pumped into the kiln from the bottom of the kiln; the fire is then extinguished only at the end of the quenching process with some water dousing from the top avoiding thus that pyrolysis gases escape unburned. Alternatively, quenching water is introduced at one side only until the water level reaches the top. |
| 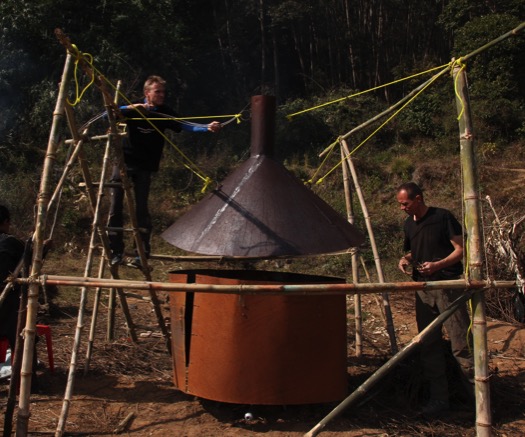 | Measurement of the gas emissions using a chimney to channel the escaping gases so that they could be measured simultaneously. |
