# Supplementary material for: Emissions and Char Quality of Flame-Curtain "Kon Tiki" Kilns for Farmer-Scale Charcoal/Biochar Production
Source: PLoS One. 2016 May 18;11(5):e0154617. doi: 10.1371/journal.pone.0154617 (PMC4871524; doi:10.1371/journal.pone.0154617)
Supplement: S2 Fig — TGA analyses of two representative biochars (BCE-soil and BCE-wood). Temperature was ramped from 25 to 950°C in 2 hours. "Gewichtsverlust" is loss of weight (both rate and overall loss), "Zeit" is time. (DOCX) [file pone.0154617.s004.docx]

**S2 Fig. TGA analyses**. TGA analyses of two representative biochars (BC_E-soil_ and BC_E-wood_). Temperature was ramped from 25 to 950 ºC in 2 hours. "Gewichtsverlust" is loss of weight (both rate and overall loss), "Zeit" is time.


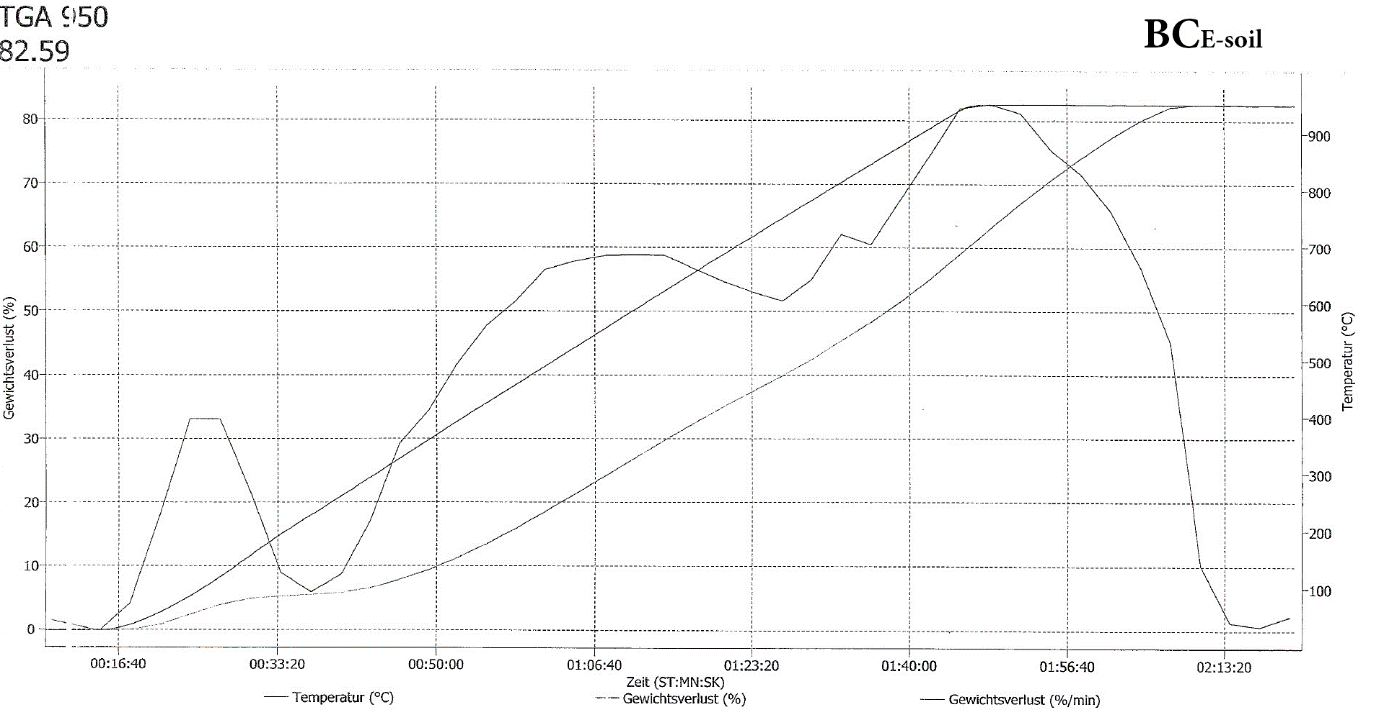


Total weight loss

Temperature

Weight loss rate


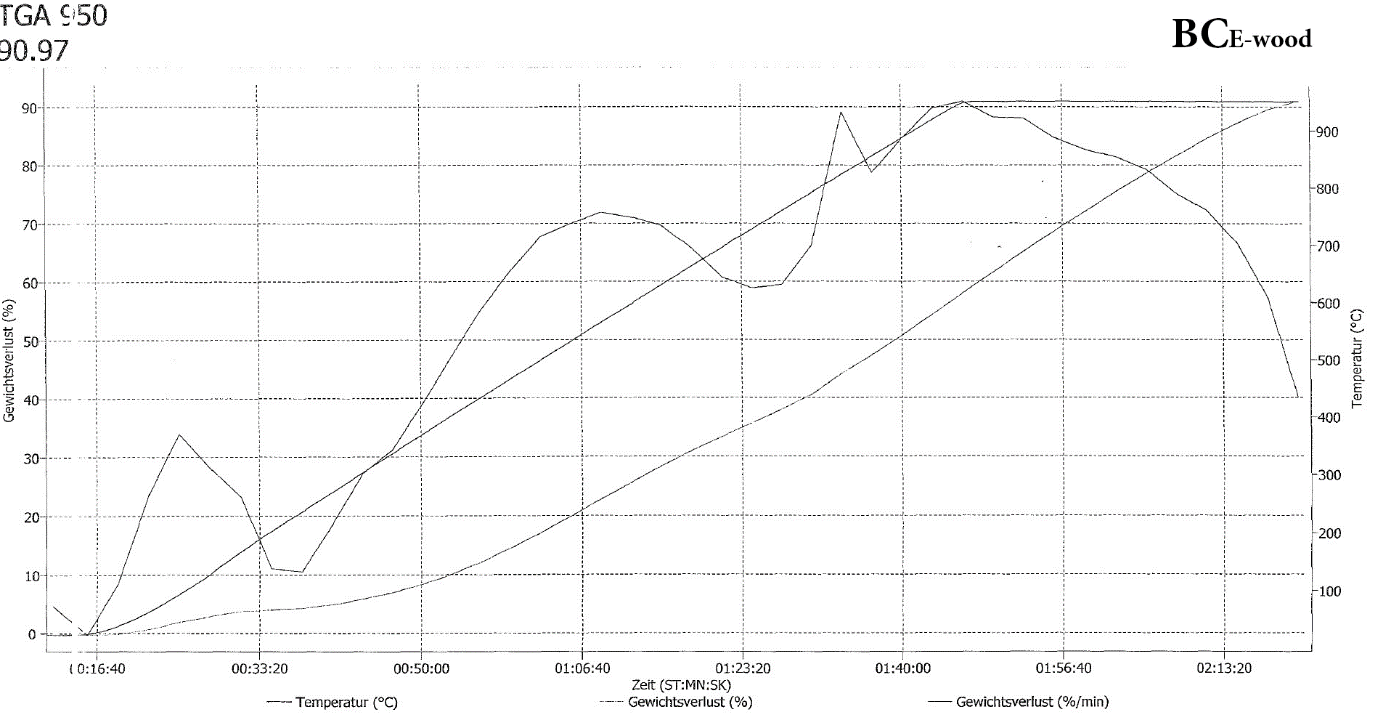


Temperature

Total weight loss

Weight loss rate
